# Identification of Potential Parkinson’s Disease Drugs Based on Multi-Source Data Fusion and Convolutional Neural Network

**DOI:** 10.3390/molecules27154780

**Published:** 2022-07-26

**Authors:** Jie Liu, Dongdong Peng, Jinlong Li, Zong Dai, Xiaoyong Zou, Zhanchao Li

**Affiliations:** 1 School of Chemistry and Chemical Engineering, Guangdong Pharmaceutical University, Guangzhou 510006, China; lemonfino@163.com (J.L.); pdd201907@163.com (D.P.); leekamlong@163.com (J.L.); 2 School of Biomedical Engineering, Sun Yat-Sen University, Guangzhou 510275, China; daizong@mail.sysu.edu.cn; 3 School of Chemistry, Sun Yat-Sen University, Guangzhou 510275, China; 4 Key Laboratory of Digital Quality Evaluation of Chinese Materia Medica of State Administration of Traditional Chinese Medicine, Guangzhou 510006, China; 5 NMPA Key Laboratory for Technology Research and Evaluation of Pharmacovigilance, Guangzhou 510006, China

**Keywords:** Parkinson’s disease, drug repositioning, convolutional neural network, multi-source data fusion

## Abstract

Parkinson’s disease (PD) is a serious neurodegenerative disease. Most of the current treatment can only alleviate symptoms, but not stop the progress of the disease. Therefore, it is crucial to find medicines to completely cure PD. Finding new indications of existing drugs through drug repositioning can not only reduce risk and cost, but also improve research and development efficiently. A drug repurposing method was proposed to identify potential Parkinson’s disease-related drugs based on multi-source data integration and convolutional neural network. Multi-source data were used to construct similarity networks, and topology information were utilized to characterize drugs and PD-associated proteins. Then, diffusion component analysis method was employed to reduce the feature dimension. Finally, a convolutional neural network model was constructed to identify potential associations between existing drugs and LProts (PD-associated proteins). Based on 10-fold cross-validation, the developed method achieved an accuracy of 91.57%, specificity of 87.24%, sensitivity of 95.27%, Matthews correlation coefficient of 0.8304, area under the receiver operating characteristic curve of 0.9731 and area under the precision–recall curve of 0.9727, respectively. Compared with the state-of-the-art approaches, the current method demonstrates superiority in some aspects, such as sensitivity, accuracy, robustness, etc. In addition, some of the predicted potential PD therapeutics through molecular docking further proved that they can exert their efficacy by acting on the known targets of PD, and may be potential PD therapeutic drugs for further experimental research. It is anticipated that the current method may be considered as a powerful tool for drug repurposing and pathological mechanism studies.

## 1. Introduction

Parkinson’s disease (PD) is the second most common chronic progressive neurodegenerative disease after Alzheimer’s disease. It has many causes and clinical manifestations [1,2,3], and the incidence of PD is gradually rising with the progress and development of society. The pathological hallmark of PD is the loss of dopaminergic neurons in the substantia nigra pars compacta and the accumulation of α-synuclein-rich intraneuronal aggregates [4,5], resulting in insufficient dopamine release and characteristic motor symptoms. Currently, the gold standard for treating this disease is oral administration of the dopamine precursor levodopa to increase dopamine production in the nigrostriatum. However, this medication has long-term adverse effects. Combination of catechol-O-methyltransferase inhibitors can reduce motor fluctuations in advanced PD [6]. Dopaminergic agonists act based on direct stimulation of postsynaptic dopaminergic receptors and are suitable for reducing motor symptoms in the early stages of the disease [7,8]. Monoamine oxidase type B inhibitors relieve symptoms by reducing the degradation of dopamine catalyzed by monoamine oxidase [9]. Meanwhile, surgery has also become a common alternative treatment due to long-term drug resistance and side effects. Deep brain stimulation is a surgical technique with high-frequency electrical stimulation for symptomatic treatment of PD [10,11]. It uses surgically implanted electrodes for the treatment of patients with motor fluctuations, retardation and other symptoms. It is not effective for dopamine resistance symptoms and can cause various side effects, such as neuropsychiatric abnormalities and cognitive dysfunction [12,13]. PD-like symptoms were significantly improved by transplanting dopaminergic neuron-rich human fetal midbrain cells into the striatum of PD patients [14,15,16]. Immunotherapy improves disease symptoms mainly by removing overexpressed α-synuclein [17,18]. Growth factors (GFs) were used for the amelioration of neurodegenerative diseases by protecting and restoring degenerating neurons and enhancing their functional activity [19]. While these treatments and drugs can control or relieve symptoms, they cannot reverse PD. Therefore, it is very urgent to find new drugs to treat PD.

New drug development has always been a time-consuming, high-risk and challenging process. Many drug candidates can not be approved by FDA and used for clinical treatment [20,21]. In contrast, drug repositioning (i.e., finding new indications for existing drugs) is the more cost- and time-effective approach, because the repositioned drug has been verified through drug and toxicology testing. It makes drug repositioning more important and becomes an alternative strategy for drug research [22,23,24]. Quantitative structure–activity relationship (QSAR) has been used to identify Parkinson’s disease-related lead compounds. For example, Khanfar et al. [25] combined extensive pharmacophore modeling and QSAR analysis to explore the structural requirements for potent Adenosine A2A antagonists, which were potential anti-Parkinson’s disease drugs. Sebastián-Pérez et al. [26] used QSAR modeling to identify LRRK2 inhibitors for Parkinson’s disease. A PD treatment drug, monoamine oxidase B inhibitors, was designed by Souza et al. [27] based on the QSAR. In addition, many drugs have also been relocated to Parkinson’s disease. For example, the tricyclic antidepressant clomipramine is utilized to treat obsessive-compulsive disorder by increasing the activity of certain chemicals in the brain, thereby improving delusions and hallucinations in PD patients with depression [28]. Gabapentin can not only treat epilepsy and certain types of nerve pain, but also directly affect the glutamate neuron system and the gamma-aminobutyric acid neuron system, reduce visual hallucinations and pain in PD patients, and does not have any adverse effects [29]. Thalidomide was originally used for the immune regulation of some cancers. It was found through research that it can improve the functional damage of nigrostriatal cells, manifested as a significant increase in dopamine, making it a potential PD adjuvant drug [30]. Additionally, the thalidomide derivative lenalidomide reduced motor behavioral deficits and improved dopaminergic fiber loss in the striatum by reducing microgliosis in the striatum and hippocampus, treating neuroinflammation in PD patients [31]. Glatiramer acetate, originally adopted to treat multiple sclerosis, can enhance central brain-derived neurotrophic factor (BDNF) activity and enhance neurogenesis, helping to resolve BDNF deficiency in PD [32]. Studies on salbutamol, originally used for bronchospasm in asthma, bronchitis, emphysema and other lung diseases, have shown that it can enhance the transport of levodopa across the blood–brain barrier, which improves the response of PD patients to levodopa [33]. However, successful candidate compounds are limited and we cannot effectively validate all potential drugs through clinical trials. Therefore, there is an urgent need for development computation method to discover new indications of existing drugs.

Recently, more and more machine learning methods have been proposed with the ability to process complex data for finding new drug indications by predicting potential drug–disease associations, drug–target interactions. Methods to study potential drug-disease associations fall into three main categories. The first category exploits similar association data between drugs and diseases to make potential association predictions. Zhang et al. [34] introduced a similarity-constrained matrix factorization method for identifying drug–disease associations based on drug features and disease semantic information. Zhang et al. [35] integrated drug features, semantic similarities of diseases, and known drug–disease associations using a non-negative matrix factorization approach. These methods usually only employed the similarity and association information between drugs and diseases to identify their relationships. With the rapid development of various omics, much information related to drugs and diseases, such as network topology, side effects, pathways, symptoms and targets, are becoming more and more abundant. This information has been gradually used in the research of drugs and diseases. The second category of methods utilizes multi-source data for association prediction. Liang et al. [36] integrated information about multiple attributes of a drug and employed a sparse subspace learning approach to predict associations. Wang et al. [37] employed graph-regularized matrix factorization to discover new indications for existing drugs. Luo et al. [38] used random walks on a constructed heterogeneous network for association prediction. However, it can be made better to consider the complex nonlinear relationship among multi-source data, rather than the only use of its basic information. The third category of methods are deep learning-based methods that deeply integrate various drug and disease data to improve prediction performance. Li et al. [39] employed drug molecular structure and disease clinical symptom information to characterize drugs and diseases, and recognized potential drug-disease associations through deep convolutional neural networks. Based on feature representations and deep neural network, Peng et al. [40] proposed a method named DTI-CNN for distinguishing drug–target interactions. Zeng et al. [41,42] constructed heterogeneous networks and identified new indications for known drugs through deep learning. These approaches have been successful, the drug relocation problem is furthermore worth for further research.

Here, a novel method was proposed to identify potential PD drugs through repositioning based on multi-source data fusion and convolutional neural networks (MSDF-CNN). Similarity networks were obtained through integration of multi-source data. The local and global topological information in the network were then obtained to characterize drug and PD-associated proteins (LProts). Diffusion component analysis was performed to reduce the dimensions of drug and LProt feature vectors, and low-dimensional features were considered as the input features for CNN model. Finally, the predicted PD drugs were further verified by molecular docking.

## 2. Results and Discussion

### 2.1. Redundancy Analysis of Dataset

**Figure 1:**
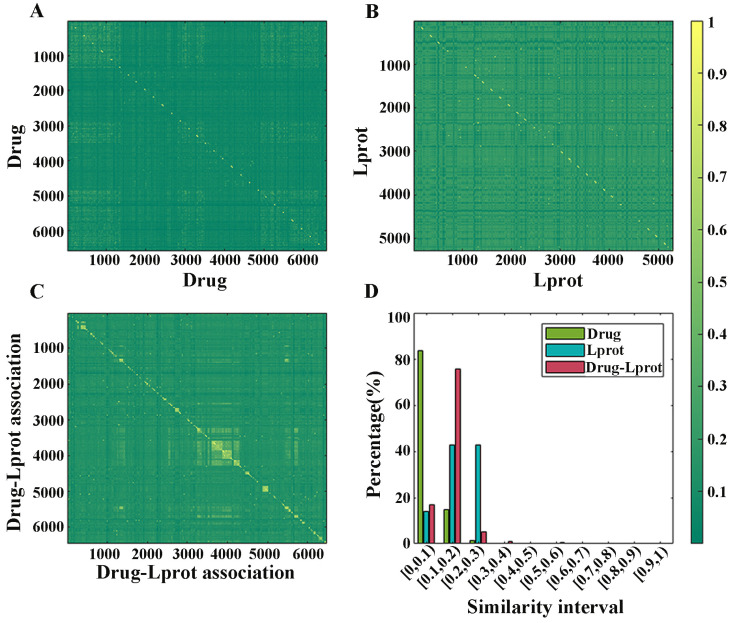
Similarity values and statistical results. (**A**–**C**) The similarity values of any two drugs, two LProts and two drug–LProt association pairs, respectively. (**D**) The statistical distribution of drugs, LProts and drug–LProt associations similarity values.

The redundancy of the dataset was analyzed by calculating the similarity values between any two drugs, two LProts (PD-associated proteins) and two drug–LProt association pairs. The similarity values and statistical results are shown in Figure 1.

For drugs, the similarity values of 83.64% are mainly concentrated in the interval [0, 0.1) (i.e., the values are greater than or equal to 0 and less than 0.1, the same below) and the intervals of [0.1, 0.9) only contain 16.36%, 14.801% in [0.1, 0.2), 1.29% in [0.2, 0.3), 0.23% in [0.3, 0.4), 0.0275% in [0.4, 0.5), 0.0092% in [0.5, 0.6), 0.0018% in [0.6, 0.7), 0.0004% in [0.7, 0.8) and 0.0001% in [0.8, 0.9); there are no similarity values in the range of [0.9, 1.0). For LProt, 13.93%, 42.71% and 42.89% of the similarity values lie in the range of [0, 0.1), [0.1, 0.2) and [0.2, 0.3), respectively. Only 0.284%, 0.0896%, 0.0158%, 0.0275%, 0.0495%, 0.0017% and 0.0019% are involved in the range of [0.3, 0.4), [0.4, 0.5), [0.5, 0.6), [0.6, 0.7), [0.7, 0.8), [0.8, 0.9) and [0.9, 1.0), respectively. For drug–LProt association pairs, the distribution of the similarity values is centralized: 16.77%, 75.66% and 5.41% are located in the range of [0, 0.1), [0.1, 0.2) and [0.2, 0.3), respectively. These results indicate that the dataset has low redundancy.

### 2.2. Optimal Feature Dimension of Diffusion Component Analysis

**Figure 2:**
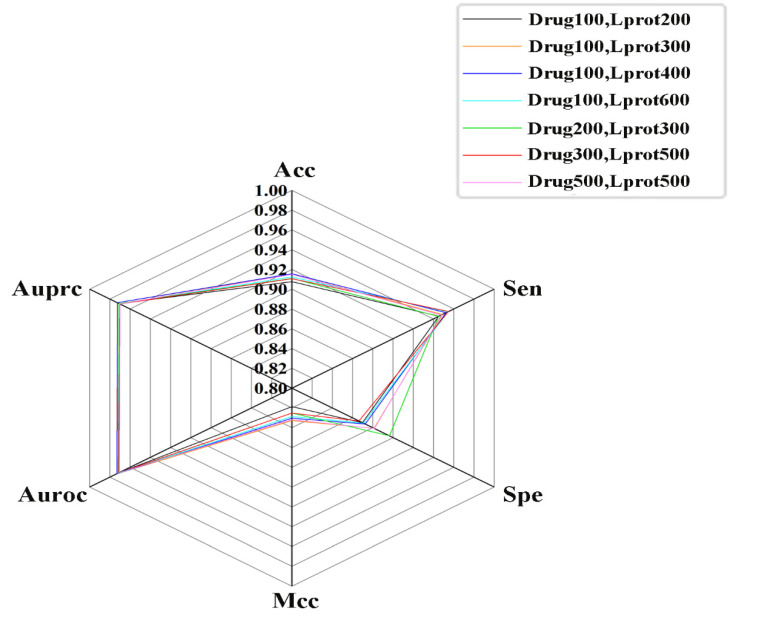
RSDs of Acc, Auroc, Auprc, Sen, Spe, Mcc.

In order to avoid over-fitting and reduce the noise of data, diffusion component analysis (DCA) method was used to reduce the dimension of drug and LProt feature vectors, respectively. The 10-fold cross-validation test was performed 10 times, and the mean and relative standard deviation (RSD%) of accuracy (Acc), area under the receiver operating characteristic curve (Auroc), area under the precision-recall curve (Auprc), sensitivity (Sen), specificity (Spe) and Matthews correlation coefficient (Mcc) are listed in Table 1 and shown in Figure 2, respectively.

From Table 1, all evaluation measures fluctuated less. When the drug feature was 100-dimensional and the LProt feature was 400-dimensional, Acc, Sen, Spe, Mcc were 91.57%, 95.26%, 87.24% and 0.8303, Auroc and Auprc reached 0.9731 and 0.9726, respectively. As shown in Figure 2, RSD% corresponding to each evaluation index were 0.33, 0.12, 0.16, 0.16, 1.72, 1.19, revealing good robustness and prediction performance for the model. Finally, the 100 dimensions of drugs and 400 dimensions of LProt were chosen as the optimal feature vector dimensions.

### 2.3. Effect of the Proportion of Positive and Negative Samples on Performance

**Figure 3:**
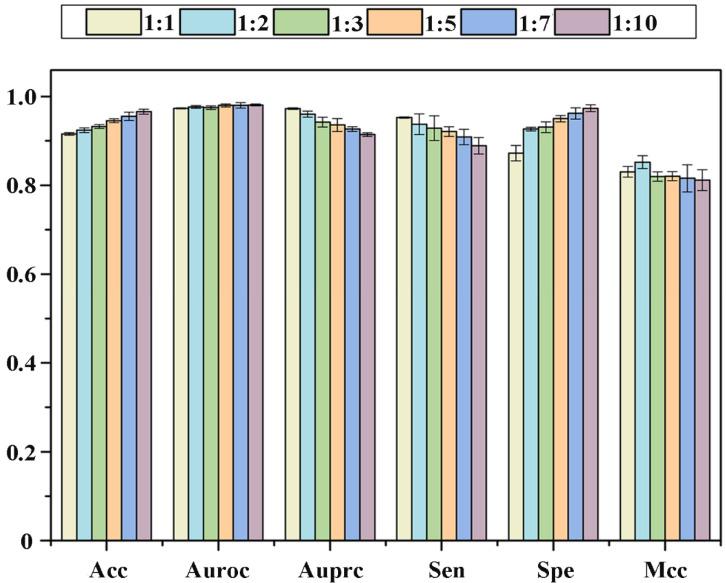
Statistical average results from datasets with various ratios between positive and negative sample.

In our study, the number of positive samples was only 6484, while the number of negative samples reached 34,871,681, which is more than 5000 times the positive samples. It is well known that the ratio between positive and negative samples may have a certain impact on the performance of the model. To explore this effect, the 6 training datasets were constructed with different ratios (1:1, 1:2, 1:3, 1:5, 1:7 and 1:10) between the positive and negative samples. The results derived from the 10-fold cross-validation test are shown in Figure 3. With the gradual increase of the number of negative samples, Acc and Spe increased significantly, Auroc improved slowly, Mcc fluctuated randomly in the range of 0.81–0.85, Auprc and Sen decreased continuously. The goal of the current study is to identify potential positive samples, which requires the model to have higher sensitivity. In addition, among these ratios of positive and negative samples, Acc, Auroc, Auprc and Sen have the smallest fluctuations when the ratio is 1:1, and RSDs% were 0.33, 0.13, 0.17 and 0.16, respectively. The optimal ratio of positive and negative samples is set to 1:1.

### 2.4. Identification Ability of New Drugs

In order to verify the recognition ability of our method for potential drugs, the 6 non-redundant datasets were constructed by setting thresholds of 0.9, 0.8, 0.7, 0.6, 0.5, 0.4. In these non-redundant datasets, the similarity of any two drug molecules is always lower than a certain threshold. Please note that the number of drugs is too small to be statistically significant when the threshold is lower than 0.4. The 10-fold cross-validation results based on various non-redundant datasets are listed in Table 2. When the threshold is reduced from 0.9 to 0.5, the fluctuation range of Acc, Sen, Spe and Mcc is very narrow and lower than 1%. The Auroc and Auprc values remain stable at around 0.9699 and 0.9691. Even though the threshold is changed to 0.4, our method still obtains Acc% of 88.49, Sen% of 94.73, Spe% of 82.24, Mcc of 0.7769, Auroc of 0.9598 and Auprc of 0.9587. These results suggest that the current method has good performance for identifying potential drugs.

### 2.5. Recognition Ability of New Targets

The identification of potential targets not only helps us to discover new therapeutic mechanism of drugs, but also find new indications. Therefore, the 6 non-redundant datasets were generated by setting thresholds of 0.9, 0.8, 0.7, 0.6, 0.5, 0.4. In these non-redundant datasets, the similarity values of any two proteins are always lower than a specific threshold. Please note that non-redundant dataset is not constructed when the threshold is set to 0.3, because very few positive samples are contained in the non-redundant dataset. The 10-fold cross-validation results are listed in Table 3.

It can be seen that Acc, Auroc and Auprc are still stable around 90.94%, 0.9669 and 0.9674, respectively, and have very narrow fluctuations (<1%) when the threshold is changed from 0.9 to 0.4. Even if the threshold is lowered to 0.4, our method can still achieve Sen of 90.84%, Spe of 92.95% and Mcc of 0.83955. These results demonstrate that our method can identify potential drug-related proteins.

### 2.6. Discriminatory Performance of Potential Drug-LProt Associations

To further verify the robustness of our method, a series of non-redundant association pair datasets were constructed according to the following steps: (1) Set a threshold. (2) Randomly select a positive drug-LProt association pair, and calculate its similarity values to other remaining positive samples. Delete the selected positive sample, if any of the similarity values are higher than the threshold; otherwise, keep it in the positive sample set. (3) Repeat step (2) until the similarity values of any two positive associations are lower than the threshold, and the obtained set is called the non-redundant positive sample set. (4) Randomly select a drug–LProt non-association pair from the negative samples set, and calculate their similarity values with each association in the non-redundant positive sample set and in the negative sample set. (5) Remove the selected association from the total negative sample, if any of the similarity values are higher than the threshold; otherwise, keep it. (6) Repeat steps (4) and (5), and establish a non-redundant negative sample set with the same sample size as the non-redundant positive sample set. Finally, the set of non-redundant positive samples and the set of non-redundant negative samples are merged into a non-redundant training dataset. Here, thresholds of 0.9, 0.8 and 0.7 were utilized to construct three non-redundant training datasets (when the threshold was set to 0.6, the non-redundant positive sample set contained too few samples to have statistical significance). The results of 10-fold cross-validation are listed in Table 4.

From Table 4, when the threshold is set to 0.9, Acc%, Sen%, Spe%, Mcc, Auroc, Auprc are 91.87, 95.58, 88.56, 0.8405, 0.9746 and 0.9764, respectively. When the threshold is changed from 0.9 to 0.7, Acc, Sen, Spe, Mcc, Auroc and Auprc only decreased by 0.12%, 0.91%, 0.45%, 0.0039, 0.0026 and 0.0023, respectively. The results show that the current method is significantly robust for identifying potential drug–LProt associations.

### 2.7. Performance Evaluation of Current Methods

**Figure 4:**
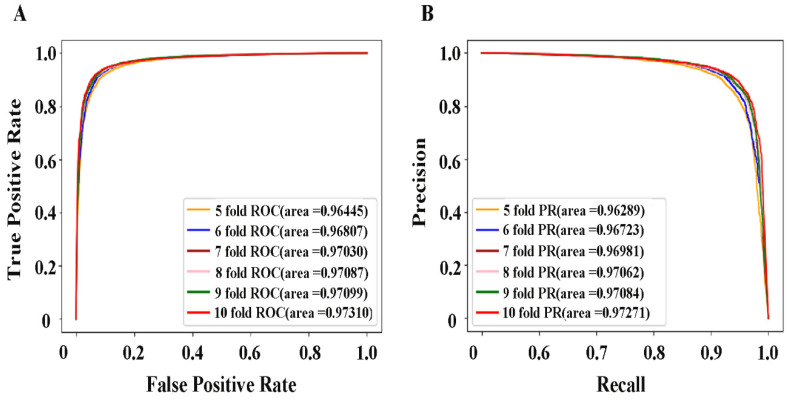
(**A**) receiver operating characteristic curve with different folds. (**B**) precision–recall curve with different folds.

In our CNN prediction model, the inputs include low-dimensional features integrated from multi-source data. Moreover, the negative samples equal to the positive samples are randomly selected, divided into training sets and test sets under different cross-validation folds. After 10 parallel experiments, the model performance was evaluated according to Auroc and Auprc. The corresponding results are shown in Figure 4, and we can observe that the 10-fold cross-validation shows the best performance and the curresponding Auroc and Auprc are 0.9731 and 0.9727, respectively. The RSD% of the evaluation indicators of the 10 training results are 0.33, 1.72, 0.16, 1.19, 0.13 and 0.17, respectively. These results indicate that the developed method can effectively capture information on drug–LProt interactions and has outstanding capabilities in identifying potential therapeutic drugs for Parkinson’s disease.

### 2.8. Comparison with Existing Methods

**Figure 5:**
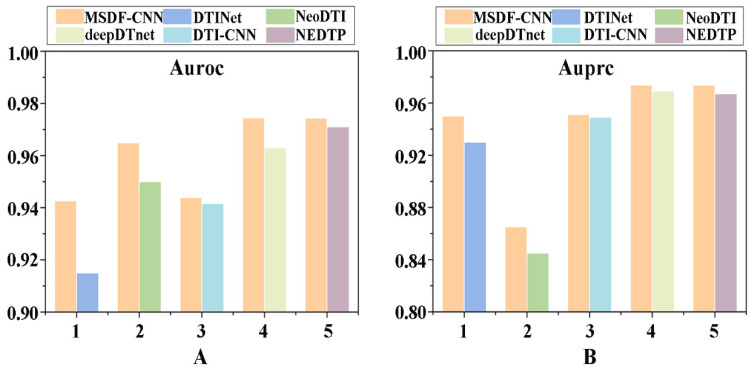
Performance comparison with existing methods. (**A**) Auroc value. (**B**) Auprc value.

A comparison is further performed with the state-of-the-art approaches. Based on integrated multiple drug and protein-related information sources, Luo et al. [43] developed a method called DTINet to predict potential drug–protein associations. The nonlinear end-to-end learning model NeoDTI was proposed by Wang et al. [44] to facilitate DTI prediction. The DTI-CNN model proposed by Peng et al. [40] obtained drug and target features in heterogeneous networks through random walks, and then used a deep neural network model to predict new drug–target interactions. deepDTnet [41] was a novel network-based deep learning method to systematically embed 15 chemical, genomic, phenotypic and cellular networks, and was used for target identification and drug repurposing under the PU learning framework. Similar to DTI-CNN model, NEDTP [45] applied random walks to the constructed heterogeneous network of drug and target similarity to extract the topology information of each node in the network as its feature vector; then build a gradient boosting decision tree model for predicting potential DTI. Based on 10-fold cross-validation, the datasets from these methods were applied to our proposed prediction model, and the corresponding average values of Auroc and Auprc through 10 parallel experiments were obtained. The results were shown in Figure 5. Compared with DTINet, NeoDTI, deepDTnet models, the Auroc and Auprc of our method are improved by 2.77%, 1.50%, 1.15% and 2.01%, 2.01%, 0.48%, respectively. For data from DTI-CNN, Auroc of 0.9469 and Auprc of 0.9512 are obtained by our model. For data from NEDTP, Auroc of 0.9710 and Auprc of 0.9737 are also achieved by the current method. In conclusion, the proposed method outperforms these five methods, and furthermore exhibited good predictive performance for identifying potential drug–LProt associations.

**Figure 6:**
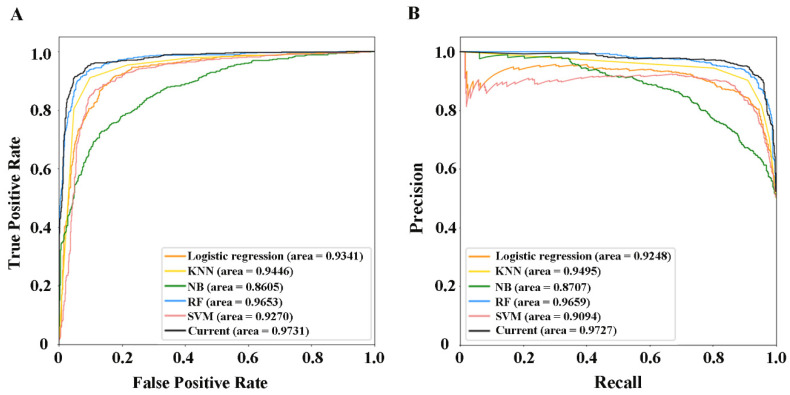
ROC and PRC curves for various methods. (**A**) ROC Curve. (**B**) PRC Curve.

Based on the 10-fold cross-validation and the benchmark dataset with ratio 1:1 of positive and negative samples, we performed a comparison with logistic regression, K-nearest neighbor (KNN), naïve Bayes (NB), random forest (RF) and support vector machine (SVM) to further demonstrate the effective of the current method. The Acc, Sen, Spe, Mcc, Auroc and Auprc are listed in Table 5, and the corresponding ROC and PRC curves are shown in Figure 6. The current method obtained the highest Acc, Sen, Mcc, Auroc and Auprc. Even though the RF acquired the highest Spe, 90.02%, the goal of the current research is to identify potential positive samples, which means the model needs to have high sensitivity, so we can conclude that the current method is more suitable for identifying potential PD drugs compared to other machine learning methods.

### 2.9. Molecular Docking

A benchmark dataset was constructed based on all positive samples and an equal number of negative samples, and was used to build the final prediction model. Then the trained model was used to predict all the unknown samples, and the corresponding correlation score value was ranked in descending order. Here, original indications of the top 10 potential drugs are listed in Table 6.

Molecular docking is a theoretical simulation method to study intermolecular interactions and predict their binding modes and affinities based on the characteristics of receptors and the interaction between receptors and drug molecules. Kim et al. [46] explored the potential therapy of hallucinogens by studying the binding mode and active state of hallucinogens to Gq-coupled 5-HT2A serotonin receptors, as well as the conformational rearrangement of receptors involved in active state transitions, which has accelerated the discovery of more selective drugs for the treatment of various neuropsychiatric disorders. Based on this study, in order to verify the reliability of the predicted results, we further selected the three top-ranked drugs to perform molecular docking simulation studies. The first is the antitumor drug topotecan, a semisynthetic derivative of camptothecin that exerts its efficacy by inhibiting type I DNA topoisomerases for the treatment of ovarian cancer [47], small cell lung cancer [48] or cervical cancer [49]. The second is loperamide [50], a nonselective calcium channel blocker that controls symptomatic relief of acute non-specific diarrhea and of chronic diarrhea associated with inflammatory bowel disease by slowing intestinal motility and by affecting water and electrolyte movement through the bowel. The third is artenimol [51], which treats uncomplicated plasmodium falciparum infections by binding to haem within the Plasmodium falciparum parasite. Pimavanserin [52,53,54,55,56,57,58,59,60,61], a drug approved by FDA in 2016 for the treatment of PD, is both a selective serotonin 2A inverse agonist and a non-dopaminergic selective serum. It can not only block HTR2A receptor, but also reduce its intrinsic activity and reach a saturated state. By binding to HTR2A receptor, it exerts its medicinal effect. The HTR2A is a G protein-coupled receptor-like protein, and functions as a receptor for various drug. Lee et al. [62] conducted a genetic association analysis of PD patients, and found that the genetic variants of HTR2A receptor may be associated with the susceptibility of impulse control and repetitive behaviors in PD patients receiving dopamine replacement therapy. Shukla et al. [63] used network pharmacology, molecular docking, and dynamic simulation methods to correlate serotonin GPCR receptors (HTR1A, HTR2A, HTR1B, HTR7 and HTR2C) common to intestinal inflammatory and neuronal diseases. Experimental results show that biologically active compounds present in W. somnifera (Withanolide A, B, E, Q and Anahygrine) interact with these receptors, which can reconstitute presynaptic and postsynaptic in neurodegenerative diseases and prevents pathogenesis and neuronal death, thereby promoting the regeneration of axons and dendrites, and then effectively preventing and/or controlling these diseases.

The HTR2A receptor has been validated as a PD target in the therapeutic target database (http://db.idrblab.net/ttd/data/target/details/t32060 (accessed on 25 March 2022), which combines with ligand to cause a conformation change, triggers signaling via guanine nucleotide-binding proteins (G proteins) and modulates the activity of downstream effectors, and signaling activates phospholipase C and phosphatidylinositol-calcium second messenger system, thereby regulating the activity of phosphatidylinositol 3-kinase and promoting the release of intracellular stored Ca(2+) ions. It affects neural activity, perception, cognition, and mood, and plays a role in behavioral regulation, including responses to anxiety situations and psychoactive substances. The main idea of our current research is to identify potential drug small molecules for Parkinson’s disease through drug–protein_1–protein_2–PD, where protein_2 is a known drug target for Parkinson’s disease, and protein_1 is related to protein_2. This is because protein_1 interacts with protein_2 and their interaction confidence score is ≥0.5, i.e., a drug can treat Parkinson’s disease through a cascade of interactions with protein_1 and protein_2. The predicted 10 top-ranked drug-protein_1–protein_2 interactions involving 5HT2A_HUMAN are shown in Table S1 from Supplementary Materials. The receptor of 5HT2A_HUMAN is presented in these association pairs, therefore, so it is selected as a PD protein receptor in molecular docking.

The three-dimensional structures of drug and HTR2A receptor were acquired from DrugBank database and predicted by Alphafold from Uniprot database, respectively. We used the pimavanserin-HTR2A complex as a positive control. Molecular docking simulations were performed by using the Autodock program, the grid center coordinates of box were set to −10.642 A, −6.476 A and −2.976 A, and Lamarckian genetic algorithm was adopted to search the docking conformation. For the complete steps of molecular docking, please refer to Table S2 from Supplementary Materials. The molecular binding energies and inhibition constants between the four drugs and the target protein are listed in Table 7.

From Table 7, the molecular binding energy between HTR2A and pimavanserin is −6.4 kcal/mol, and the inhibition constant reaches 20.49 μM. However, the predicted molecular binding energies and the corresponding inhibition constants among the topotecan, loperamide, artemisinol and HTR2A were −7.96 kcal/mol, −7.76 kcal/mol, −7.65 kcal/mol and 1.47 μM, 2.05 μM, 2.46 μM. These results are lower than those of positive control, revealing that the three drugs may be candidates for the PD.

**Figure 7:**
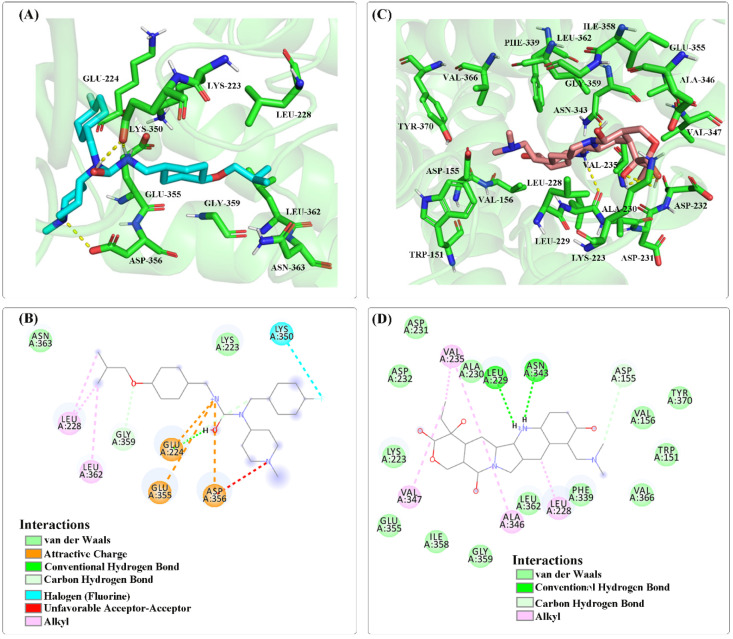
Visualization of the docking results of small molecule drugs (Pimawanserin, Topotecan) and PD target (5-HT2A). (**A**,**B**) Pimavanserin docked to receptor proteins as well as their interactions (Positive Control) (**C**,**D**) Topotecan docked to receptor proteins and their interactions.

In addition, the ligand–protein binding modes were also visualized between the drugs of pimavanserin, topotecan and target protein by using DS visualization software. As illustrated in Figure 7, pimavanserin mainly has van der Waals interaction with HTR2A receptor (residues Asn363, Lys223), attractive charges with three residues of Glu224, Glu355, Asp356, and carbon–hydrogen bond interaction with residue Gly359. Meanwhile, alkyl interactions also exist in the drug small molecule and residues of Leu228 and Leu362. There are van der Waals forces between predicted topotecan and 13 amino acid residues (residues Asp231, Asp232, Lys223, Glu355, Ile358, Gly359, Leu362, Phe339, Val366, Trp151, Tyr370, Val156, Ala230) and hydrogen bond interactions with two residues (Leu229, Asn343). The results also revealed that topotecan has carbon–hydrogen bond interaction with residue Asp155, alkyl interactions with residues of Val235, Val347, Ala346 and Leu228. Therefore, the two small molecule drugs have van der Waals forces with residue Lys223, and are also involved in an alkyl interaction with the Leu228 residue. HTR2A is a certified PD target; therefore, we speculate that topotecan may play a role in the treatment of PD and some of its side effects by acting on different sites of the HTR2A receptor.

## 3. Materials and Methods

### 3.1. Collection and Processing of Data

ATC is the abbreviation of the anatomical, therapeutic and chemical classification of drugs, which is formulated and regularly published by The WHO Collaborating Centre for drug statistics methodology. We retrieved drug information (ATC, enzymes, Smiles, targets) from the DrugBank, protein sequence information from the UniProt, drug side effects from the SIDES, protein pathway information from the CTD, and protein–protein interaction (PPI) information from the HIPPIE, respectively. For drugs, the drugbank ID were used as the drug ID, and the adjacency matrix were used to represent the relationship between the drug and the target, side effects, enzymes, ATC. Finally, the collected drug meets these conditions: the molecular fingerprint can be calculated by smiles, and it can be characterized by at least one of target protein, side effect, enzyme, and ATC. For proteins, Uniprot AC was used as its ID, and proteins that can be sequenced are retained, the relationship between proteins and pathways is represented by adjacency matrix. For the PPI, interactions with a correlation value of 0 were removed. Moreover, the self-interactions, repeated interactions, proteins without UniProt ID and/or sequence information were also deleted. Finally, we obtained 6587 drugs with 4828 drug–enzyme associations, 755,165 drug–side effect associations, 4636 drug–ATC associations and 15,504 drug–target interactions; 16,330 proteins with 353,550 PPI. In addition, we also obtained 157 PD targets and 30 known PD drugs from the TTD database. Based on the collected PPI and PD targets, the 5295 PD-associated proteins (LProt) were screened out by setting interaction confidence score (a high interaction confidence score means that two proteins are more likely to interact.) greater than or equal to 0.5. Moreover, 13,947 KEGG pathways corresponding to LProt were also considered as characterization data. Finally, based on the adjacency matrix, 6484 drug–LProt interactions including 6587 drugs and 5295 LProt were obtained by integrating collected various data and information. For detailed steps of data collection and processing, please see Table S3 from Supplementary Materials. The statistical and detailed information of the drugs and proteins are listed in Table 8 and Tables S4 and S5 from Supplementary Materials, respectively. The final collected datasets include 6484 positive samples and 34,871,681 (6587 × 5295 − 6484) negative samples.

### 3.2. Characterization of Drugs and LProt

The drug chemical structure similarity network was constructed by calculating MACCS molecular fingerprints similarity based on the Jaccard similarity coefficient. The LProt sequence similarity network was generated by protein sequence alignment based on the Smith–Waterman algorithm [43]. Execute the Jaccard similarity algorithm [64] separately for each correlation matrix and interaction matrix to obtain drug–enzyme similarity matrix, drug–side effect similarity matrix, drug–target similarity matrix, drug–ATC similarity matrix, LProt–pathway similarity matrix. The drug–side effect correlation matrix is employed as an example to detail how to construct the drug–side effect similarity matrix, in which each row and column corresponds to a drug and a side effect respectively. The corresponding element is set to 1 if a drug is associated a side effect. Otherwise, it is 0. The Jaccard similarity coefficient is a measure of the similarity between two drugs and is defined as follows:

$$S(A,B)=\frac{\left|A\cap B\right|}{\left|A\cup B\right|}$$

The original adjacency matrix is a description of the relationship between a single row and a column node, and the Jaccard similarity coefficient calculation is based on two adjacent row vectors of the original adjacency matrix. The similarity matrix *S* represents the similarity between all features of each drug or the protein node and the column node, and the elements S*_i,j_* represent the similarity between the *i*-th and *j*-th rows in the original adjacency matrix. In our study, five drug similarity networks and two LProt similarity networks were used for the characterization of drugs and LProt, respectively. Please refer to Table S3 from Supplementary Materials for a detailed description of drug and LProt characterization.

### 3.3. Extraction and Selection of Feature

For drug–side effect similarity matrix, we firstly define the transition matrix *W* based on the PageRank algorithm, which represents the transition probability between different nodes. Secondly, random surfing model [65] with restart probability α at the initial node of each iteration is utilized to capture topology information. The probability that it returns to the original vertex and restarts the process is 1−*α*, the formula is as follows:

$$P_{k}=\alpha P_{k-1}W+(1-\alpha )P_{0}$$

where *P_k_* is a row vector, in which nth entry indicates the probability of reaching the nth vertex after *k* steps of transitions. The *P*_0_ is the initial one-hot vector with the value of the *i*th entry being 1 and all other entries being 0.

Based on the probabilistic co-occurrence (PCO) matrix generated by the random surfing model, we calculate a shifted positive pointwise mutual information (*PPMI*) matrix by following Bullinaria and Levy [66] to express the co-occurrence probability among nodes. This method generates new network representations by decomposing the co-occurrence matrix. The *PPMI* matrix formula is as follows:

$$PPMI=\max\left[\log\frac{M(m,n)\times \sum_{m}^{N_{d}}\sum_{n}^{N_{t}}M(m,n)}{\sum_{m}^{N_{d}}M(m,n)\times \sum_{n}^{N_{t}}M(m,n)},0\right]$$

where *M* is the original co-occurrence matrix, *N_d_* is the number of drugs, and *N_t_* is the number of LProt. We assign each negative value to be 0.

For other similarity matrices, the steps mentioned above were also performed. So far, the seven PPMI matrices for drug and LProt features representation were obtained, such as drug-structure (D_1_), drug-enzyme (D_2_), drug-side effects (D_3_), drug-target (D_4_), drug-ATC (D_5_), LProt-sequence (P_1_) and LProt-pathway (P_2_). Finally, the concatenating 5 drug features and 2 LProt features were utilized to characterize drug–protein association pair with 32935-dimensional drug feature and 10590-dimensional LProt feature.

The drug and LProt feature vectors were the high-dimensional and sparse, which usually not only increase the computational burden but also lead to poor prediction performance. Therefore, the diffusion component analysis (DCA) method [43,67] was adopted to reduce its dimensionality. In this process, the dimensions of the drug and LProt features are reduced from 32,935 to 100 and 10,590 to 400 by minimizing the difference between the diffusion distributions of individual networks and the corresponding model distributions. The learned low-dimensional feature vectors encode the relational properties, association information, and topological context of each node in the heterogeneous drug–LProt network.

### 3.4. Construction and Evaluation of Models

Inspired by the successful application of convolutional neural networks in classification tasks [68], a one-dimensional convolutional neural network (CNN) model is constructed to identify potential PD drugs. The architecture and parameters of the model are listed in Table 9. In the CNN model, Adam algorithm was utilized to optimize parameters. In addition, the initial learning rate, epochs and sample number of each batch were set to 0.01, 80 and 512, respectively.

To evaluate the performance of the model, *Acc*, *Spe*, *Sen* and Matthews correlation coefficient (*Mcc*) are employed. Meanwhile, area under the receiver operating characteristic curve (Auroc) and area under the precision–recall curve (Auprc) are also utilized to assess prediction performance. *Acc*, *Spe*, *Sen* and *Mcc* can be calculated according to the following equations:

$$\begin{array}{l} A\mathrm{cc}=\frac{TP+TN}{TP+TN+FP+FN}\times 100\%\\ Spe=\frac{TN}{TN+FP}\times 100\%\\ Sen=\frac{TP}{TP+FN}\times 100\%\\ Mcc=\frac{TP\times TN-FP\times FN}{\sqrt{\left(TP+FN)\times (TN+FN)\times (TP+FP)\times (TN+FP\right)}} \end{array}$$

where *TP* (true positive) is the number of positive samples that are correctly predicted as positive samples, *FP* (false positive) is the number of negative samples that are incorrectly predicted as positive samples, *TN* (true negative) is the number of negative samples that are correctly predicted to be classified as the number of negative samples, *FN* (false negative) is the number of wrongly predicted positive samples as negative samples.

**Figure 8:**
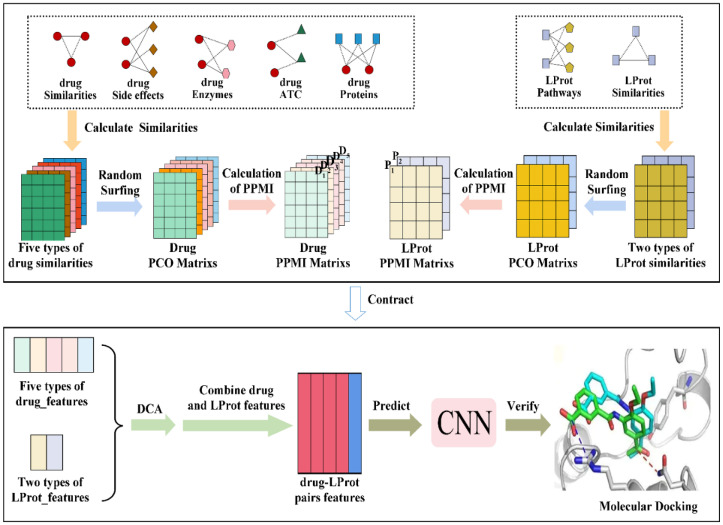
MSDF-CNN workflow.

The flowchart of the current method is illustrated in Figure 8, and the detailed steps are follows.

(1) Set a threshold based on the PPI network and known PD targets to screen out LProts (PD-associated proteins) with high correlation.
(2) Construct multiple drug and LProt networks according to multi-source data and characterized by similarity networks.
(3) Obtain high-dimensional features of drugs and LProt by capturing global and local topological information in the network.
(4) Employ diffusion component analysis to reduce dimensionality and obtain low-dimensional and rich features for drug and LProt.
(5) Construct a convolutional neural network model to predict potential association pairs.
(6) Evaluate and verify the prediction and application performance of the developed method by using the 10-fold cross-validation test and molecular docking research, respectively.

## 4. Conclusions

In this study, multi-source similarity networks of drugs and PD-associated proteins were used to characterize drugs and LProt, and topological information in the network was further obtained for the characterization of drug–LProt interactions. The convolutional neural network was utilized to build a classification model to predict potential treatments for Parkinson’s disease. The proposed method has robustness and good prediction performance. In addition, through molecular simulations, the reliability of the potential therapeutic drugs was further verified. In conclusion, our current work provides a new avenue for research to discover new therapeutic drugs for Parkinson’s disease, and has important implications for the study of drug repositioning methods and the pharmaceutical industry.

## Figures and Tables

**Table 1:** Average values of Acc, Sen, Spe, Mcc, Auroc, Auprc.

Dimension		Acc (%)	Spe (%)	Sen (%)	Mcc	Auroc	Auprc
Drug	LProt						
100	200	90.76	94.54	86.98	0.8187	0.9709	0.9709
100	300	91.51	94.88	88.13	0.8328	0.9730	0.9729
100	400	91.57	95.26	87.24	0.8303	0.9731	0.9726
100	600	91.25	95.43	87.08	0.8287	0.9721	0.9708
200	300	91.08	94.51	89.65	0.8252	0.9711	0.9710
300	500	91.06	95.49	86.63	0.8251	0.9715	0.9702
500	500	91.50	94.77	88.15	0.8320	0.9706	0.9702

**Table 2:** 10-fold cross-validation test results on different non-redundant datasets.

Threshold	Acc (%)	Sen (%)	Spe (%)	Mcc	Auroc	Auprc
0.9	91.57	95.27	87.24	0.8304	0.9732	0.9727
0.8	91.81	95.21	88.41	0.8387	0.9735	0.9726
0.7	91.34	94.92	87.76	0.8294	0.9708	0.9691
0.6	90.74	95.41	86.07	0.8193	0.9699	0.9691
0.5	90.11	94.61	85.61	0.8065	0.9658	0.9654
0.4	88.49	94.73	82.24	0.7769	0.9598	0.9587

**Table 3:** 10-fold cross-validation test results on different non-redundant drug datasets.

Threshold	Acc (%)	Sen (%)	Spe (%)	Mcc	Auroc	Auprc
0.9	91.92	94.85	89.05	0.8408	0.9726	0.9728
0.8	90.23	94.32	86.13	0.8087	0.9666	0.9661
0.7	90.24	94.37	86.11	0.8090	0.9667	0.9654
0.6	90.73	93.62	87.85	0.8171	0.9652	0.9658
0.5	90.59	94.20	86.98	0.8151	0.9660	0.9654
0.4	91.90	90.84	92.95	0.8395	0.9645	0.9692

**Table 4:** 10-fold cross-validation results of the non-redundant drug–LProt association datasets.

Threshold	Acc (%)	Sen (%)	Spe (%)	Mcc	Auroc	Auprc
0.9	91.87	95.58	88.56	0.8405	0.9746	0.9764
0.8	91.84	95.58	88.25	0.8397	0.9757	0.9760
0.7	91.75	94.67	88.11	0.8366	0.9720	0.9741

**Table 5:** Comparison results of 10-fold cross-validation with different methods.

	Acc (%)	Sen (%)	Spe (%)	Mcc	Auroc	Auprc
Logistic regression	86.51	86.84	86.19	0.7303	0.9341	0.9248
KNN	87.29	94.40	80.16	0.7534	0.9446	0.9495
NB	77.62	71.46	83.82	0.5569	0.8605	0.8707
RF	90.57	91.12	90.02	0.8114	0.9653	0.9659
SVM	86.92	88.12	85.72	0.7386	0.9270	0.9094
Current	91.57	95.27	87.24	0.8304	0.9731	0.9727

**Table 6:** Top ten drug information.

Number	Drug	Indication
1	Topotecan	Treat ovarian cancer, small cell lung cancer or cervical cancer.
2	Loperamide	Control nonspecific and chronic diarrhea caused by inflammatory bowel disease or gastroenteritis.
3	Artenimol	Treatment of artemisinin derivatives and the antimalarial agent Plasmodium falciparum infection.
4	Mitotane	Treatment of inoperable adrenal cortical tumors; Cushing’s syndrome.
5	Estramustine	The palliative treatment of patients with metastatic and/or progressive carcinoma of the prostate.
6	Quercetin	A flavonol widely distributed in plants. It is an antioxidant, like many other phenolic heterocyclic compounds.
7	Nortriptyline	A tricyclic antidepressant used to treat major depressive disorder and also to treat chronic pain and other conditions.
8	Bacitracin	Topical preparations for acute and chronic topical skin infections.
9	Minocycline	Treatment of inflammatory lesions of acne vulgaris.
10	Doxepin	A psychotropic agent with antidepressant and anxiolytic properties.

**Table 7:** Molecular docking results of pimavanserin, loperamide, topotecan, artemisinol and PD target (HTR2A).

Ligand	Target Protein	Binding Energy (kcal/mol)	Inhibition Constant (μM)
Pimavanserin	HTR2A	−6.4	20.49
Loperamide		−7.76	2.05
Topotecan		−7.96	1.47
Artenimol		−7.65	2.46

**Table 8:** The detail information of the drugs and proteins.

Information	Number	Sources
drug–chemical structure	6587	DrugBank Database
drug–ATC	4636	
drug–enzyme	4828	
drug–target	15,504	
drug–side effect	755,165	SIDES Database
PPI	353,550	HIPPIES Database
PD targets	157	TTD Database CTD Database Uniprot Database DrugBank Database
PD drugs	30	
PD associated targets (LProt)	5295	PPI PD targets
LProt–pathway	13,947	CTD Database
LProt–sequence	5295	Uniprot Database
drugs	6587	DrugBank Database

**Table 9:** The framework and parameters of convolutional neural network.

Layer	Size
Input	500*1
Convolutional	4 filters with 5*1, stride 1*1
ReLU	-
Convolutional	8 filters with 10*1, stride 1*1
ReLU	-
Max-Pooling	2*1, stride 2*1
ReLU	-
Fully connected	256, dropout = 0.5
Sigmoid	-
Classification	2

## Data Availability

The datasets and predicted results are available at https://github.com/lemonfino/MSDF-CNN (accessed on 4 April 2022).

## Supplementary Materials

The following supporting information can be downloaded at: https://www.mdpi.com/article/10.3390/molecules27154780/s1. Table S1: The predicted top-ranked 5HT2A-HUMAN-related drug-LProt associations; Table S2: Complete steps for molecular docking; Table S3: Detailed steps for data collection, processing and characterization; Table S4: Detailed drug related data; Table S5: Detailed protein related data.

## Sample Availability

Samples of the compounds are not available from the authors.
