# Supplementary material for: Identification of Potential Parkinson’s Disease Drugs Based on Multi-Source Data Fusion and Convolutional Neural Network
Source: Molecules. 2022 Jul 26;27(15):4780. doi: 10.3390/molecules27154780 (PMC9369596; doi:10.3390/molecules27154780)
Supplement: Supplementary file 1 [file molecules-27-04780-s001.zip › Table S2.pdf]

Supplementary Materials 2:

Complete steps for molecular docking

1. Preparation of Receptors and Ligands

Step 1: Download the pdb file of the 3D structure of small molecule drugs from the DrugBank database (https://go.drugbank.com/), and the pdb file of the 3D structure of the protein predicted by Alphafold from the Uniprot database (https://alphafold.ebi.ac.uk/).

Step 2: Open the AutoDockTools software, import the pdb file of the protein, remove water molecules, add full hydrogen, and export it as a pdbqt file (set as acceptor). Import the pdb file of the drug, add full hydrogen, set it as a ligand, and save it as a pdbqt file.

2.Grid settings

Step 1: Open receptor and ligand in pdbqt format in Grid → Macromolecule and Set MapTypes respectively, check if the ligand position is too close to the active pocket, and remove it.

Step 2: Grid → Grid Box, and then adjust the position, shape, and size of the Box, so that the cube box covers (Binding sites: amino acid residues Leu228, Lys223, Leu362, Glu224, Val235, Val347, Ala346, Leu229, Asn343, etc.). In the upper left corner of the Box setting window file → quit save current, and finally Grid → Output → save as grid.gpf.

Step 3: In the Run → AutoGrid → window, select the third file path as grid.gpf that was just saved, then launch, and wait patiently for the small window to close automatically.

3. Docking

Step 1: 1. Docking → Macromolecule → Rigid → Select protein.pdbqt.

2. Docking → Ligand → choose → select drug.pdbqt → select Ligand → Accept

Step 2: 1. Docking → Search Parameters → Genetic Algorithm... → Accept

2. Docking → Docking Parameters... → Accept

3. Docking → Output → GA(4.2) → Save as dock.dpf. (note the manual entry of the suffix)

Step 3: Run → Autodock → The third option is to select dock.dpf → Launch → Wait for the small window to close automatically → Edit → Delete → all.

4. View the docking result

Step 1: Analyze → Dockings → Open just docked the automatically saved dlg file, and then Analyze → Macromolecule → Open to display the relevant ligands and receptors.

Step 2: Analyze → Comformation → Load to see all the indicators of Pose.

Step 3: 1. Analyze → Comformation → Play, ranked by energy. Can view the appearance of each Pose. Then, select the penultimate icon on the right side of the player, tick the first item in the window, and can see the detailed indicators of each Pose.

Step 4: Set Play Options → Write Complex → Save as pdbqt format, which can be opened with Discovery Studio.
