# Supplementary material for: Identification of Potential Parkinson’s Disease Drugs Based on Multi-Source Data Fusion and Convolutional Neural Network
Source: Molecules. 2022 Jul 26;27(15):4780. doi: 10.3390/molecules27154780 (PMC9369596; doi:10.3390/molecules27154780)
Supplement: Supplementary file 1 [file molecules-27-04780-s001.zip › Table S3.pdf]

Supplementary Materials 3:

Detailed steps for data collection, processing and characterization

For drugs: drug-chemical structure, drug-ATC, drug-enzyme, drug-target, drug-side effect:

Step 1. Download drugs and their structure information of smiles from the DrugBank database, and get 11168 drug-chemical structure pairs (Supporting information 3-1, drug-chemical structure pairs).

Step 2. The MACCS (Molecular Access System) molecular fingerprint descriptor (166 dimentions) of each drug were calculated using RDKit software, delete 18 drugs (Supporting information 3-1, delete 18 drugs) that can not calculate MACCS molecular fingerprint descriptor, and finally obtain a drug matrix of size 11150*166.

Step 3. Download the corresponding ATCs, enzymes, targets, and side effects of 11150 drugs from the DrugBank database and SIDES database, respectively. Obtain 5115 drug-ATC associations (drugs:3224, ATC:4589) (Supporting information 3-1, drug-ATC associations), 5080 drug-enzyme associations (drugs:1699, enzymes:379) (Supporting information 3-1, drug-enzyme associations), 17016 drug-target associations (drugs:5713, targets:2878) (Supporting information 3-1, drug-target associations), and 787681 drug-side effect associations (drugs:1309, side effects:15890) (drug-side effect associations).

Step 4. Based on the collected data, construct drug-enzyme and drug-side effect adjacency matrices. For drug-ATC associations, the first three levels (anatomy, therapeutics, pharmacology) are chosen to calculate its adjacency matrix. For drug-target associations, the remaining 16979 drug-target association pairs (drug: 5710, target: 2867) are obtained after delete the target that can not calculate the global alignment of protein sequence. Then, generate its adjacency matrix.

Step 5. Integrate all data so that the collected drugs meet these conditions: the molecular fingerprints can be calculated by smiles, and characterized by at least one of ATC, enzymes, targets, and side effects. Finally, the data are obtained including 6587 drugs, 4828 drug-enzyme associations, 755165 drug-side effect associations, 4636 drug-ATC associations and 15504 drug-target interactions.

Step 6. Based on adjacency matrix of drug-chemical structure, drug-ATC, drug-enzyme, drug-target, and drug-side effect, the corresponding similarity matrices were calculated by using Jaccard coefficient, and the corresponding 5 drug similarity matrices were finally obtained.

For PD-associated protein (LProt): LProt-pathway, LProt-sequence:

Step 1. Download protein-protein interaction pairs (PPIs) from the HIPPIE database, delete self-interactions, repeat interactions, and proteins without uniprot id and/or sequence information, and get 353550 PPIs (including 16330 proteins) (Supporting Information 3-2, PPIs).

Step 2. Download PD-related targets and PD-related drugs from the CTD database, TTD database, Uniprot database and DrugBank database. After delete duplication, 30 PD drugs and 157 PD targets are obtained (Supporting Information 3-2, PD drugs and PD targets).

Step 3. Based on the retrieved PPI and PD targets, 5295 PD-associated proteins (LProt) (including 11166 PPI-associated pairs) are screened out by setting an interaction confidence score greater than or equal to 0.5 (Supporting Information 3-2, PD-associated proteins).

Step 4. Download 5295 protein sequences corresponding to LProt from the Uniprot database. To obtain protein-pathway associations, the gene-pathway associations from the CTD database are mapped by Uniprot database. Then, an adjacency matrix are obtained containing 13947 LProt-pathway associations (Supporting Information 3-2, LProt-pathway associations).

Step 5. Based on the 5295 LProt's sequences , global alignment algorithm was used to calculate the similarity matrix. Based on the LProt-pathway adjacency matrix, the Jaccard algorithm was used to obtain the similarity matrix. Finally, we got 2 LProt similarity matrices.
